# Seasonal Stability of the Circadian Rhythm in Patients with Type I Myocardial Infarction

**DOI:** 10.31083/j.rcm2507259

**Published:** 2024-07-10

**Authors:** Juan-Carlos Díaz-Polanco, Carlos Tejada-González, Amanda Leandro-Barros, Antonio Ruiz-Saavedra, Elvira García-de-Santiago, Joaquín Alonso-Martín, Alberto García-Lledó, Manuel Martínez-Sellés

**Affiliations:** ^1^Department of Cardiology, Hospital Universitario Príncipe de Asturias, 28805 Madrid, Spain; ^2^Department of Medicine and Medical Specialties, University of Alcalá, 28805 Madrid, Spain; ^3^Código Infarto Madrid, Health Department of the Government of the Autonomous Community of Madrid, 28013 Madrid, Spain; ^4^Department of Cardiology, Hospital Universitario Gregorio Marañón, CIBERCV, Universidad Europea, Universidad Complutense, 28040 Madrid, Spain

**Keywords:** circadian rhythm, acute myocardial infarction, seasonal, primary angioplasty

## Abstract

**Background:**

A circadian rhythm of myocardial infarction has been described but there is little data on its relation with seasons and months.

**Methods:**

From June 2013 to June 2018, we analyzed the alerts for acute ST-segment elevation myocardial infarction (STEMI) in a Spanish region with 6.64 million inhabitants, universal health coverage, and an organized STEMI reperfusion network. We selected those patients which an identifiable culprit plaque.

**Results:**

We recruited 6765 cases of STEMI due to type I acute myocardial infarction (type-I AMI), with mean age of 63.2 years (range 17–101, standard deviation [SD] 13.7), 5238 were males (77.4%) and 2801 (41.9%) were 65 years or older. The hourly distribution followed a fixed pattern in all months, with most of the events occurring between 6:00 AM and 4:00 PM, a peak at approximately 01:00 PM and a valley between 10:00 PM and 06:00 AM. No significant difference was found when comparing the mean time to first medical contact between July (the month with more daylight hours) and December (the month with shortest days). No significant differences were found between male and female patients, or between patients aged 65 years or older and younger patients. There was a close correlation between the number of events per month and the number of events occurring during the day (6 AM to 6 PM, r = 0.988, *p* = 0.001) and during the night (6 PM to 6 AM, r = 0.944, *p* < 0.001), with different slopes of the regression lines (*t*-test, *p* < 0.001), so that the difference between day-night occurrences increased with the total incidence.

**Conclusions:**

There is a circadian pattern in the presentation of STEMI that is not influenced by sex and age. The different incidence of STEMI at different times of the year does not affect the circadian pattern in terms of the shape of the curve or the mean time of presentation, although diurnal events increase more than nocturnal events, suggesting that triggers are most likely to act during vulnerable periods as determined by a circadian-based rhythm.

## 1. Background

Circadian rhythms are a natural phenomenon in which oscillations occur in various biological processes, including the sleep cycle [1]. These rhythms are controlled by two types of clocks: a central one located in the suprachiasmatic nucleus of the hypothalamus [2], and others present in different tissues at the genetic level [3]. The existence of a diurnal rhythm affecting the incidence of an acute myocardial infarction (AMI) has been known since the middle of the last century [4]. Numerous studies have shown an increase during the morning [4, 5, 6, 7, 8]. Regional, cultural and perhaps ethnic variations in this rhythm have also been described [9, 10]. The main determinant of the rhythm set by the central nervous system is thought to be light [11]. Light affects the frequency of neuronal discharges and modifies both the expression of circadian genes [12] and the activity of the autonomic nervous system, thereby altering fundamental factors of circulatory regulation such as blood pressure, endothelial response, and platelet aggregation [11]. The activation of the autonomic nervous system during waking hours may explain the increased incidence of AMI in the morning. There are other factors that influence the regulation of circadian rhythms and can cause circadian disruption, in particular changes in physical activity [13], stress, and mealtimes. In mammals, there is a daily rhythm of increased activity that precedes the availability of food, called the food-entrainable oscillator or food-anticipatory activity [14]. Disruption of the circadian rhythm by these factors modifies circulatory variables [15] and could act as a trigger for cardiovascular events such as AMI, both through changes in heart rate and vascular reactivity or through rupture of the atheroma plaque [11]. Previous studies have demonstrated a diurnal cycle in AMI, but there is limited evidence in relation to type I infarctions due to atheroma plaque rupture [16] and the potential relation of circadian patterns with seasons and months.

The aim of this study is to observe possible variations in the hourly incidence of AMI throughout the year, based on the hypothesis that the main determinant of the circadian rhythm affecting the incidence of AMI is the habits of the subjects, rather than the light cycles. In order to analyze a unique pathophysiological phenomenon, only cases of type I ST-segment elevation myocardial infarction (STEMI) were included.

## 2. Methods

### 2.1 Population

The study population includes STEMI patients prospectively enrolled in the “*Código Infarto Madrid*” registry [17] in whom a culprit plaque was confirmed during cardiac catheterization for primary angioplasty. Data were collected between June 2013 and June 2018, the date of the last independent audit. “*Código Infarto Madrid*” is a public program aimed at coordinating reperfusion therapy for STEMI in the Community of Madrid, a region in Spain with full public health coverage and a population of approximately 6.7 million inhabitants. The program has been previously described in detail [18]. The researchers received an anonymized version of the official registry, including age, sex, date, time of first medical contact, and coronary angiography findings.

### 2.2 Definitions

The STEMI alert implies activation of a system but not a final diagnosis. To eliminate cases without a definitive diagnosis of STEMI, we included only those in which a culprit plaque was identified on angiography. As the time of symptom onset depends on the perception and memory of patients who are sometimes critically ill, the time estimate is mainly based on the time to the first medical contact. Our study uses this time, in hours and minutes, as recorded by the regional emergency system. Patients for whom the time to first medical contact was not detailed were excluded.

### 2.3 Statistics

Continuous variables are summarized as mean plus/minus standard deviation (SD). Discrete variables are expressed as percentages. To analyze the possible effect of the length of daylight on the circadian rhythm of an AMI occurrence, cases were grouped by month of the year, irrespective of the year in which the events occurred. Thus, for statistical analysis, all events that occurred in January of each of the five years were considered together. Since a possible effect of sunshine on the time of onset of STEMI during the summer has been described [19], the events recorded each month were divided into two groups, considering as diurnal those that occurred between 6 AM and 6 PM and as nocturnal those that occurred between 6 PM and 6 AM. The average time of emergency activation was estimated for each month. The possible differences between these mean times for the twelve groups were tested by analysis of variance (ANOVA), and the possible difference between the mean time of emergency system activation during the months with longer and shorter daylight hours (July and December, respectively) was tested by Student’s *t*-test for unrelated samples. Pearson’s correlation test was used to analyze bivariate linear correlations between continuous variables. A *p*-value of less than 0.05 was considered statistically significant. The statistical package SPSS® V 29.0.0.0 (IBM Corp, Armonk, NY, USA) was used for analysis.

## 3. Results

**Fig. 1.**
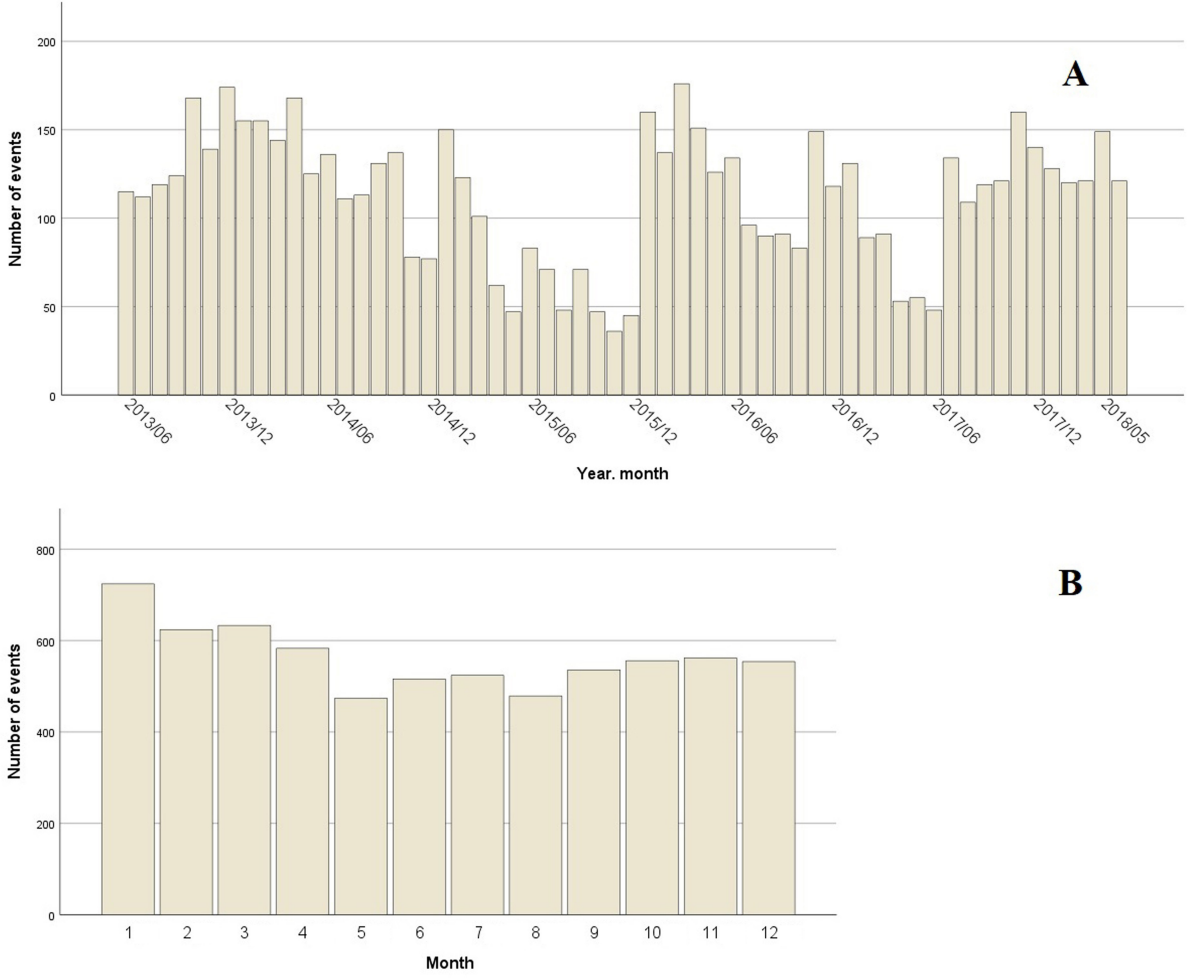
**Number of events by month.** (A) Bar chart showing the number of episodes of type I acute myocardial infarction per month, from June 2013 to May 2018. (B) Bar chart showing the number of episodes of type I acute myocardial infarction grouped by month, between 2013 and 2018.

During the 5-year study period, the registry recorded 6765 cases of STEMI with an identified culprit plaque. Of these, 5238 (77.4%) were men. The mean age of the sample was 63.2 years (range 17–101, SD 13.667), of which 2801 (41.9%) were 65 years or older. Events were more frequent in winter and less common in summer (Fig. 1A), with January, February and March being the months with the highest incidence (Fig. 1B).

**Fig. 2.**
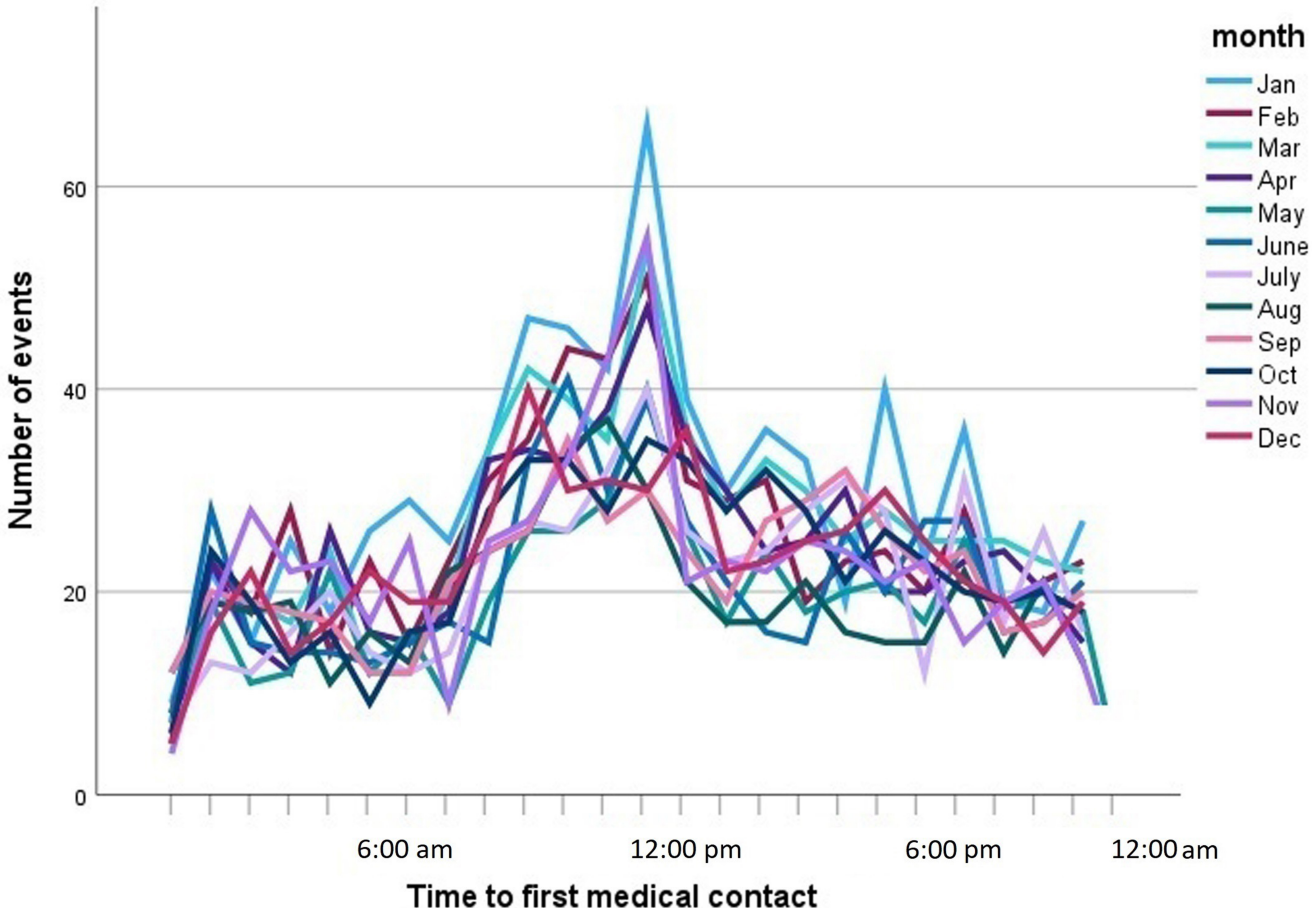
**Line graph showing the number of episodes of type I acute myocardial infarction by time to first medical contact.** Each line corresponds to one month of the year.

The number of daily events was similar across the seven days of the week, with a range from 13.3% of episodes occurring on Fridays and 15.3% on Sundays (*p* not significant). STEMI tended to occur 20 minutes later on Sundays than on weekdays (12:20:59 PM vs 12:01:36 PM, *p* not significant). The hourly distribution of infarctions followed a fixed daily pattern throughout the year, and were more frequent between 6 AM and 4 PM, with a peak around 1 PM and a valley between 10 PM and 6 AM (Fig. 2).

**Fig. 3.**
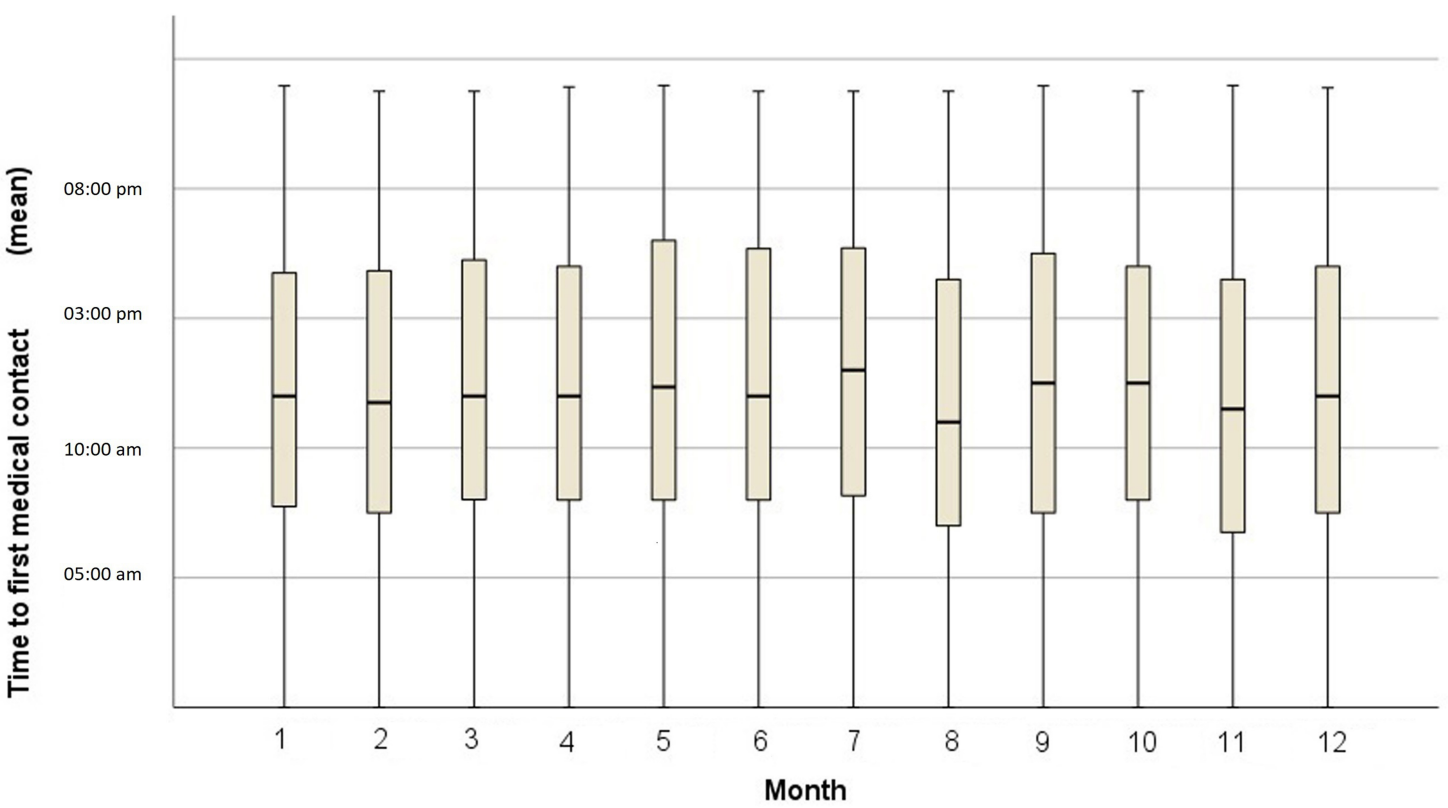
**Box plot showing the mean time to first medical contact by month**.

**Fig. 4.**
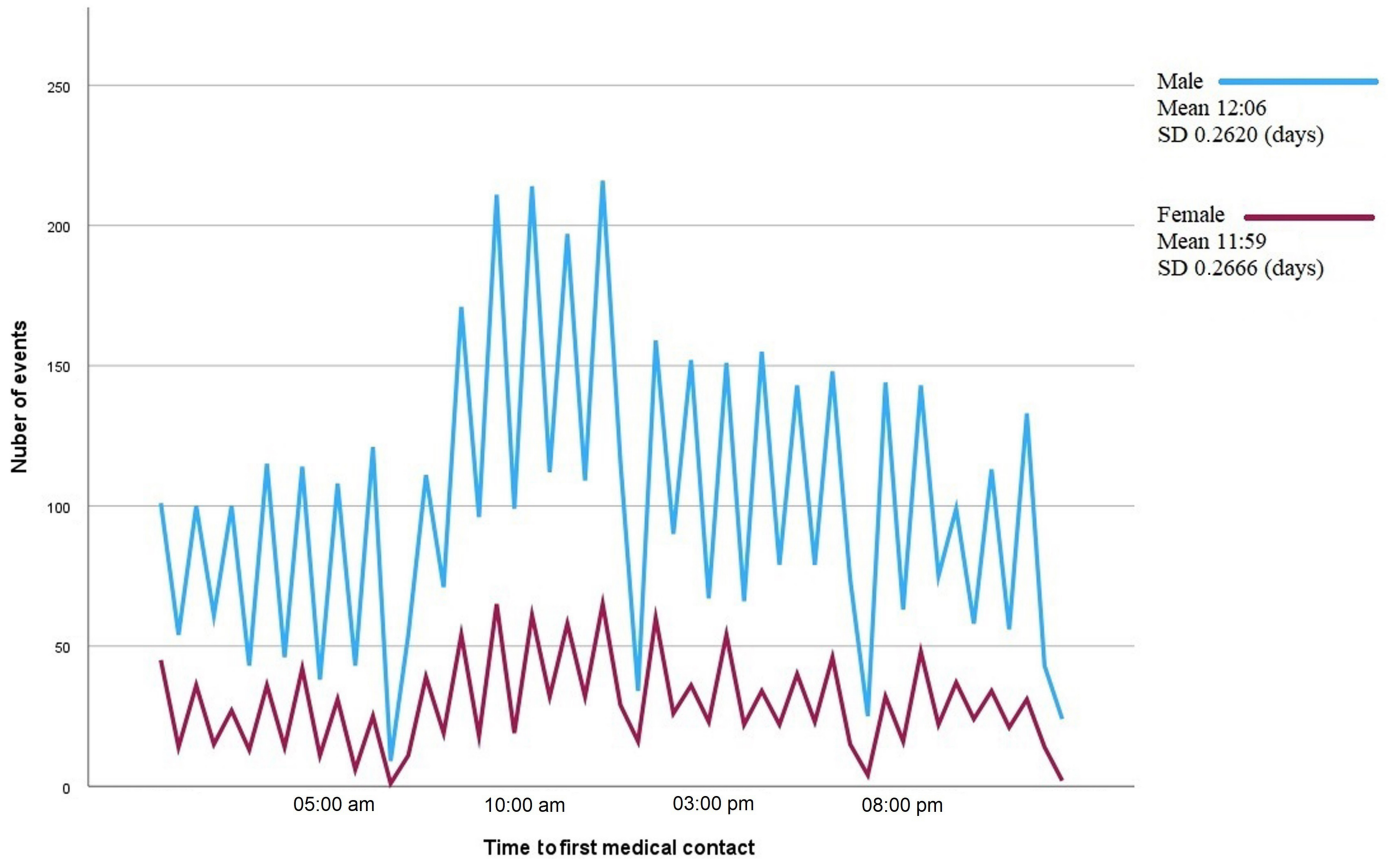
**Line graph showing the number of episodes of type I acute myocardial infarction by time to first medical contact.** Each line corresponds to one sex. The mean time to first medical contact and the standard deviation (SD) are shown on the right-hand side of the figure.

**Fig. 5.**
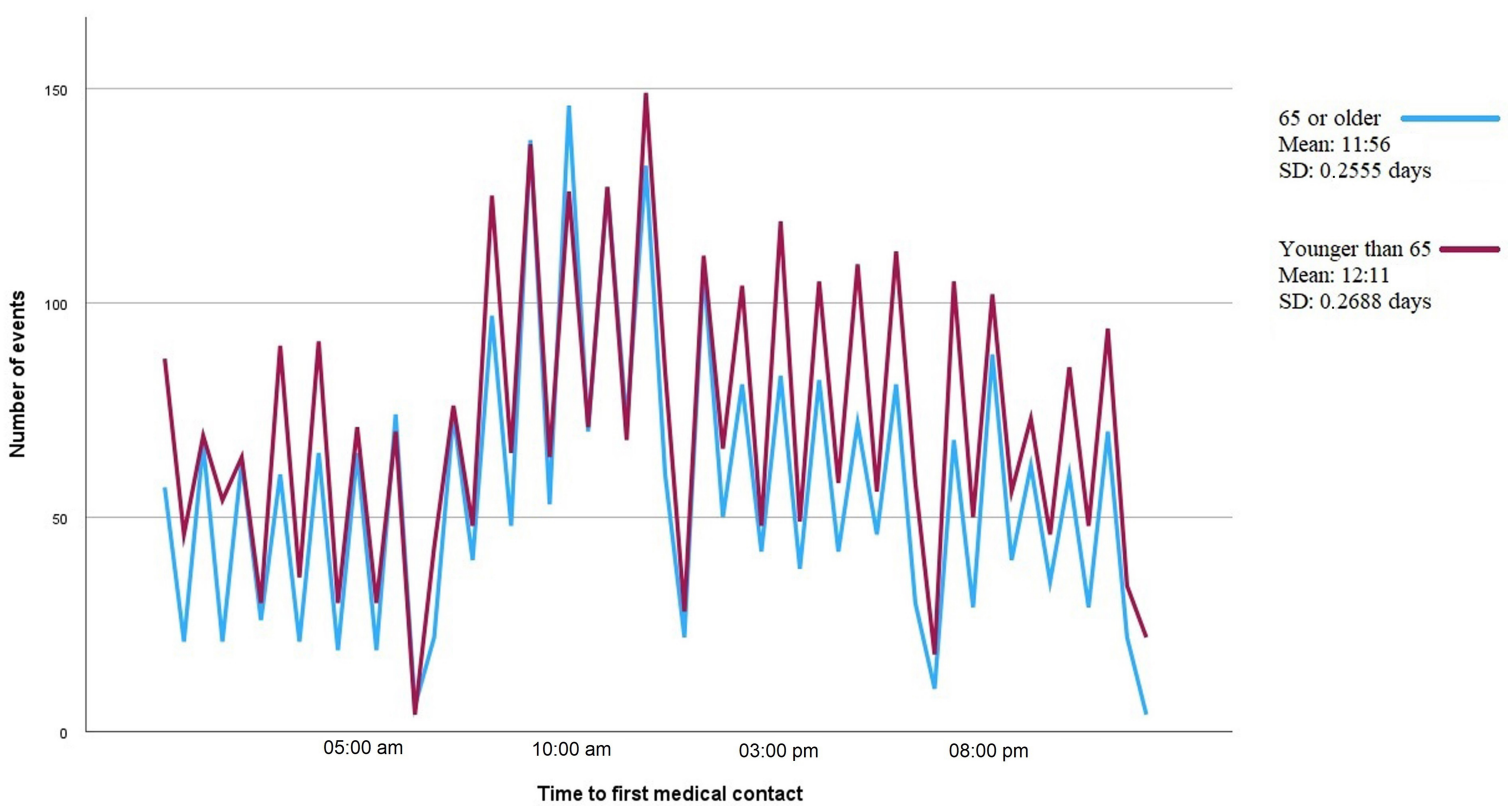
**Line graph showing the number of episodes of type I acute myocardial infarction by time to first medical contact. **Each line corresponds to patients grouped by age, 65 years and older or younger than 65 years. The mean time to first medical contact and the standard deviation (SD) are shown on the right-hand side of the figure.

The mean time of presentation of STEMI did not change throughout the year, always around 12:00 PM (*p* = 0.134, ANOVA, Fig. 3). When the mean time to system activation was compared between months with more and less sunlight (July and December, respectively), no significant difference was found, although a tendency to delay the time was observed in the month of July (12:40 PM vs 12:02 PM, *p* = 0.098). No differences in presentation were found when men and women were analyzed separately, nor when patients older and younger than 65 years were compared (Figs. 4,5).

**Fig. 6.**
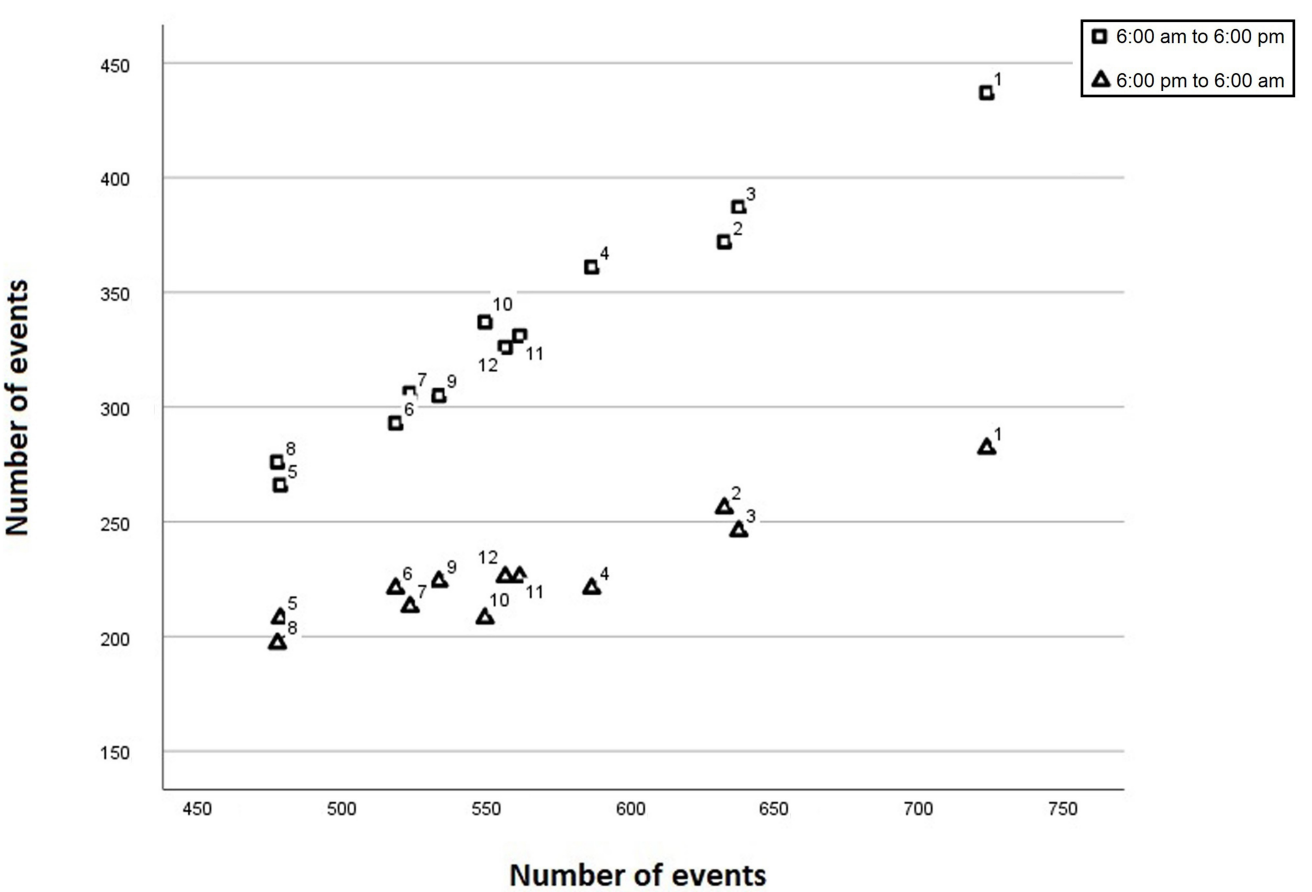
**Scatterplot showing the correlation between the total number of events (y-axis) and the number of events from 6 AM to 6 PM (squares) and from 6 PM to 6 AM (triangles).** The number next to each symbol corresponds to the month of the year.

There was a close correlation between the total number of events per month and the number of events attended during the periods defined as daytime (6 AM to 6 PM, r = 0.988, *p* = 0.001) and nighttime (6 PM to 6 AM, r = 0.944, *p* < 0.001). We found a clear difference in the slope of the regression lines as the total incidence increased, with a maximum in January and a minimum in May and August (*t*-test, *p* < 0.001, Fig. 6), so that the difference between day-night events increased with the total incidence.

## 4. Discussion

Our study shows the existence of a circadian rhythm in type I STEMI. The incidence of these events is highest around midday and lowest at night, with no variation in this pattern throughout the months of the year. Winter months have the highest incidence. When the incidence increases, it does so both in the morning and at night, but mainly during the day.

The existence of a circadian rhythm in the incidence of AMI has been described in several studies since the middle of the last century [4] and also affects other clinical manifestations closely related to acute coronary syndrome, such as sudden death [20]. However, the different manifestations of acute coronary syndrome are due to different pathophysiological phenomena, which may follow different rhythms. Whereas vasospasm tends to be a predominantly nocturnal phenomenon [21], thrombotic phenomena tend to occur preferentially in the morning [22]. This may contribute to the finding of different patterns in different studies. A study of 7032 cases of acute coronary syndrome showed a bimodal pattern with morning and evening peaks [23], which may be explained by the inclusion of a heterogeneous group in which predominantly thrombotic and predominantly spastic phenomena are mixed. The same reasons may have caused the bimodal patterns found in studies in which AMI was defined solely by elevations in biomarkers [7], thus including infarcts with potentially different pathophysiological etiologies [16]. Studies based on a population selected for treatment with primary angioplasty usually show a single peak [24]. This is the case in our study, where patients were selected on the basis of the presence of a plaque responsible for type I STEMI.

The fundamental role of light as a regulator of the circadian rhythm [11, 12] may suggest that variations in the number of daylight hours during the seasons should affect the presentation of AMI. We are aware of only one other study that has considered this possibility [19]. Cannistraci *et al*. [19] described a possible shift in the incidence of AMI towards the evening hours during the summer months, which they relate to sunlight hours and serum vitamin D concentration. Using the same time intervals and considering all months of the year, our study shows the same circadian rhythm pattern in the distribution of the peak incidence and mean alert activation time. When the incidence of infarction increases, it does so both during the day and at night, but much more during the day. This phenomenon, which to our knowledge has not been previously described, explains the apparent nocturnal shift in incidence during the months with fewer infarctions. Our interpretation is that, in a relatively fixed circadian pattern, infarct triggers such as influenza and cold weather [25] find a patient more vulnerable during the day due to increased physical activity, feeding rhythms, and cycles of sympathetic and cortisol activation [11, 13, 14, 15], although they also cause an increase in events during the night.

### Limitations

Our study does not record the actual time of onset of the acute coronary event, but rather the time at which the patient sought medical attention. The exact time of the event is difficult to determine because it depends on the patient’s perception and memory. For this reason, STEMI care systems use the time to first medical contact as a reference, which is an objective value [17]. The time from symptom onset to first medical contact is different for each care network, depending on the dispersion of the system and the design of the network. In our case, the median is estimated to be 88 minutes [17], which would anticipate the curves presented in this paper by about an hour and a half. However, some studies have shown that the time taken by patients to seek care is shorter during the day [26].

The Madrid STEMI registry does not record cardiovascular risk factors, previous events, or treatments. The lack of such data does not allow a detailed characterization of the population studied, which would allow the identification of potential differences. Of the various risk factors, hypertension seems to be the most clearly associated with circadian rhythms [27]. A different prevalence of hypertension in the sample or different treatments could affect the results. The same applies to the use of beta-blockers. In the intravenous streptokinase in acute myocardial infarction (ISAM) study [5], it was observed that patients on beta-blockers did not have an increase in the incidence of myocardial infarction in the morning. However, it is important to keep in mind the aim of our study, which is to evaluate the possible variations in circadian rhythm during the seasons and months of the year. As the treatments we are considering are chronic and are not usually modified in a way that is conditioned by the months or seasons of the year, the effect of treatment modifications could cause confusion, but not during a specific period. The number of patients and the inclusion over a five-year period may help to mitigate the effect of these limitations.

## 5. Conclusions

There is a circadian pattern in the presentation of type-I STEMI, that is not influenced by sex and age. The different incidence of STEMI at different times of the year does not affect the circadian pattern in terms of the shape of the curve or the mean time of presentation, although diurnal events increase more than nocturnal events, suggesting that triggers are most likely to occur during vulnerable periods determined by a circadian-based rhythm.

## Data Availability

The datasets used and/or analyzed during the current study are available from the corresponding author on reasonable request.
